# Assessing response in endoscopy images of esophageal cancer treated with total neoadjuvant therapy via hybrid-architecture ensemble deep learning

**DOI:** 10.3389/fonc.2025.1590448

**Published:** 2025-05-06

**Authors:** Peng Yuan, Meichen Liu, Hangzhou He, Liang Dai, Ya-Ya Wu, Ke-Neng Chen, Qi Wu, Yanye Lu

**Affiliations:** ^1^ State Key Laboratory of Holistic Integrative Management of Gastrointestinal Cancers, Beijing Key Laboratory of Carcinogenesis and Translational Research, Endoscopy Center, Peking University Cancer Hospital and Institute, Beijing, China; ^2^ Key Laboratory of Carcinogenesis and Translational Research (Ministry of Education), Endoscopy Center, Peking University Cancer Hospital and Institute, Peking University School of Oncology, Beijing, China; ^3^ Institute of Medical Technology, Peking University Health Science Center, Peking University, Beijing, China; ^4^ Department of Biomedical Engineering, College of Future Technology, Peking University, Beijing, China; ^5^ National Biomedical Imaging Center, Peking University, Beijing, China; ^6^ Key Laboratory of Carcinogenesis and Translational Research (Ministry of Education), The First Department of Thoracic Surgery, Peking University Cancer Hospital and Institute, Peking University School of Oncology, Beijing, China

**Keywords:** esophageal cancer, endoscopy, total neoadjuvant therapy, pathological complete response, deep learning

## Abstract

**Background and Aims:**

Esophageal cancer (EC) patients may achieve pathological complete response (pCR) after receiving total neoadjuvant therapy (TNT), which allows them to avoid surgery and preserve organs. We aimed to benchmark the performance of existing artificial intelligence (AI) methods and develop a more accurate model for evaluating EC patients’ response after TNT.

**Methods:**

We built the Beijing-EC-TNT dataset, consisting of 7,359 images from 300 EC patients who underwent TNT at Beijing Cancer Hospital. The dataset was divided into Cohort1 (4,561 images, 209 patients) for cross-validation and Cohort 2 (2,798 images, 91 patients) for external evaluation. Patients and endoscopic images were labeled as either pCR or non-pCR based on postoperative pathology results. We systematically evaluated mainstream AI models and proposed EC-HAENet, a hybrid-architecture ensembled deep learning model.

**Results:**

In image-level classification, EC-HAENet achieved an area under the curve of 0.98 in Cohort 1 and 0.99 in Cohort 2. In patient-level classification, the accuracy of EC-HAENet was significantly higher than that of endoscopic biopsy in both Cohorts 1 and 2 (accuracy, 0.93 *vs*. 0.78, P<0.0001 and 0.93 *vs*. 0.71, P<0.0001).

**Conclusion:**

EC-HAENet can assist endoscopists in accurately evaluating the response of EC patients after TNT.

## 1 Introduction

Esophageal cancer (EC) has a high incidence and mortality rate compared to other types of malignant tumors (1). Surgery is the primary treatment for locally advanced EC. Although complications and mortality have decreased with technological progress, 0.4% to 2.2% of patients die during the perioperative period (2). Existing studies have shown that patients who achieve pathological complete response (pCR) after neoadjuvant chemotherapy have better overall survival and disease-free survival than those without pCR (3). pCR is defined as the absence of any tumor residue in both the primary tumor site and lymph nodes. However, due to the low pCR rate in the era of chemotherapy, all patients have typically undergone further surgical intervention, regardless of their response to neoadjuvant therapy. With the development of systemic therapy based on chemotherapy and immunotherapy, the high pCR rate has made organ preservation possible (4, 5). The phase III clinical trial - SANO - published in 2023 indicated that patients who achieve CR after total neoadjuvant therapy (TNT) may choose the Watch and Wait, not surgery (6). Additionally, many more phase III clinical trials are underway. Therefore, there is an enormous demand for accurately identifying pCR after TNT to optimize treatment strategies and avoid unnecessary surgeries.

Endoscopy is an important method for evaluating the tumor residue at the primary tumor site after TNT, and detecting tumor residue in lymph nodes may require other methods, such as fine needle aspiration (FNA), CT or PET-CT. The preparation before the SANO trial - pre-SANO showed that bite-on-bite biopsies, endoscopic ultrasonography, and fine-needle aspiration could evaluate locoregional responses, and PET-CT can assess interval metastases (7). However, even using bite-on-bite biopsies combined with fine-needle aspiration, 10% of TRG 3–4 and 23% of TRG 2–4 tumors were still missed (7, 8). Further research indicates that the number of biopsies, rather than performing deep biopsies, is crucial for improving detection accuracy (9). However, the number of biopsies cannot be infinitely increased due to factors such as bleeding after biopsy. Given these challenges, new methods are needed to enhance the accuracy of identifying pCR during endoscopic examination.

Artificial intelligence (AI), particularly deep learning, has demonstrated significant advantages in improving the diagnostic accuracy of EC. First, AI could achieve satisfactory diagnostic accuracy in early EC. In a study involving 218 patients with early EC and 7,976 images, AI showed a sensitivity of 0.9, specificity of 0.89, positive predictive value (PPV) of 0.77, and negative predictive value (NPV) of 0.97 (10). More than that, AI also outperforms endoscopists with less than 15 years of experience (11). Second, AI could assist endoscopists in enhancing diagnostic accuracy. A study by Waki et al. demonstrated that incorporating AI assistance significantly improved the sensitivity of endoscopists in diagnosing EC, particularly in less experienced endoscopists (12). Therefore, we hope that AI can improve the accuracy of response assessment after TNT. Regrettably, 2 previous studies used only 98 and 123 patients for training and validation, and the accuracy was only 0.81 and 0.70, respectively, but still higher than endoscopists 0.66 (13, 14).

In this study, we hope to demonstrate that the deep learning model could achieve an accuracy superior to endoscopic biopsy, and ultimately, enhance the accuracy of endoscopic biopsy with the assistance of a deep learning model to provide an ethical and technical basis for making non-surgical decisions. To achieve these, we first developed a large dataset called the Beijing-EC-TNT dataset, comprising 7,359 endoscopic images from 300 EC patients at Beijing Cancer Hospital. Using this dataset, we evaluated various mainstream AI models and identified three key insights for designing more accurate endoscopic AI models: pretraining, efficient local features, and robust global features. Based on these findings, we built EC-HAENet, a hybrid-architecture ensemble deep learning model that demonstrated superior performance compared to endoscopic biopsy in evaluating patient responses to TNT.

## 2 Materials and methods

### 2.1 Study design and participants

**Figure 1:**
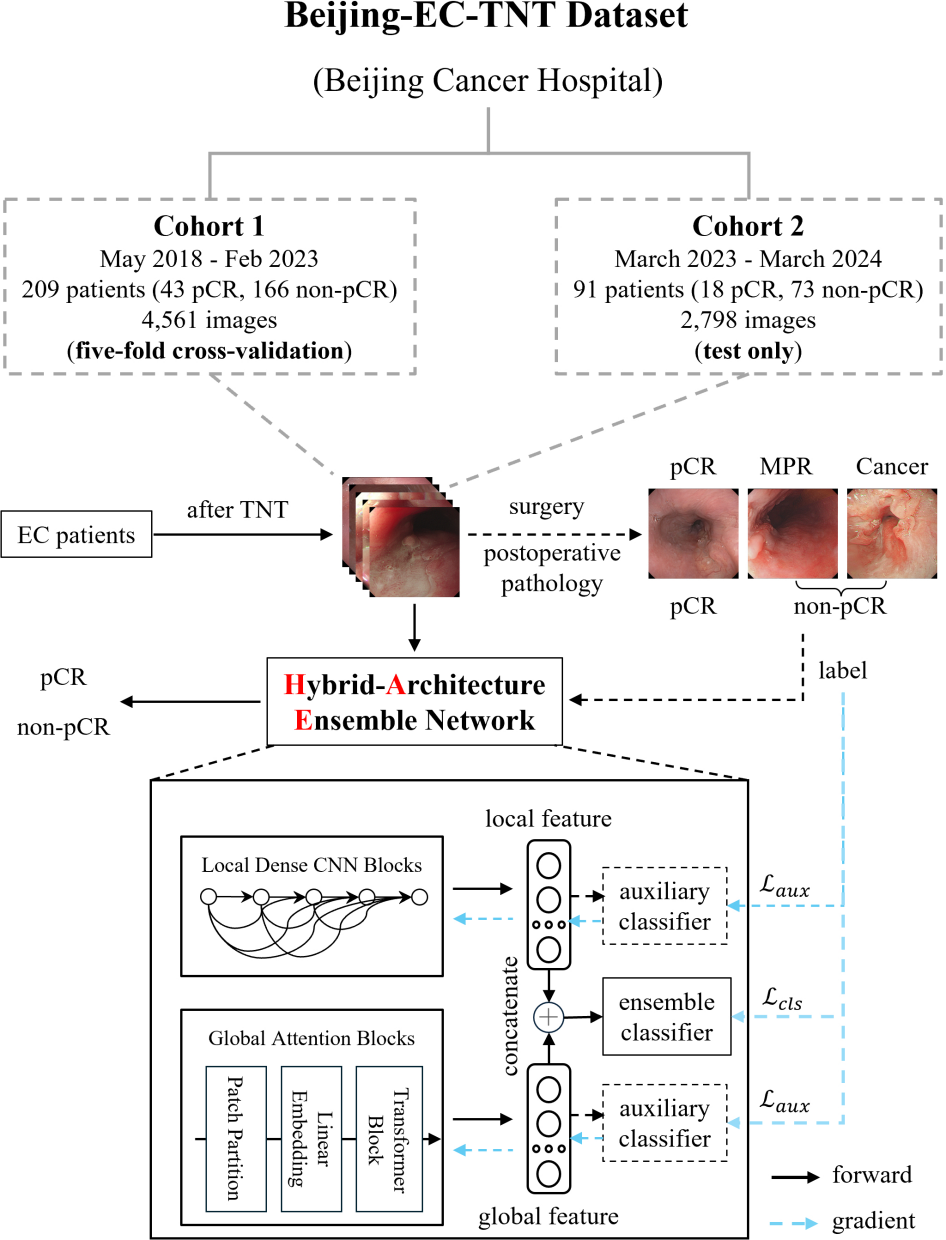
Dataset construction and model architecture of EC-HAENet. The dashed line indicates the auxiliary module during the training phase, and the blue arrow indicates the direction of the gradient update of the loss function. EC, esophageal cancer; TNT, total neoadjuvant therapy; pCR, pathological complete response; MPR, major pathological response.

This was a retrospective study, approved by the Peking University Cancer Hospital Ethics Committee (2025KT33) and conducted according to the principles of the Declaration of Helsinki. All patients were informed and consented to be enrolled in the study. We conducted a retrospective multi-cohort study using white-light endoscopic images from Beijing Cancer Hospital. As shown in **Figure 1**, our Beijing-EC-TNT dataset contains two temporally independent cohorts. Cohort 1 included patients from May 2018 to February 2023, while Cohort 2 included patients from March 2023 to March 2024. The study included patients with pathologically confirmed esophageal squamous cell carcinoma or adenocarcinoma and excluded patients with rare malignant tumors such as neuroendocrine carcinomas, sarcomas, and melanomas. All examinations were performed with high-definition gastroscopes (GIF-H290, GIF-HQ290, GIF-H260 [Olympus, Tokyo, Japan] or EG-760Z, EG-760R, EG-L600ZW7, EGL600WR7, and EG-580R7 [Fujifilm, Tokyo, Japan]).

All the patients underwent TNT, which included chemotherapy, chemoimmunotherapy, or systemic therapy combined with radiotherapy. After the final TNT, all patients underwent gastroscopy performed by various senior endoscopists to evaluate response for TNT, and certain patients underwent biopsies. We saved images from the gastroscope for training and evaluating AI models. Finally, all the patients underwent surgery. Surgically resected EC tumors were staged according to the 8th edition of the TNM staging system stipulated by the American Joint Committee on Cancer. Each patient and their corresponding endoscopic images were labeled as either pCR or non-pCR based on postoperative pathology results. Furthermore, patients in the non-pCR group were further classified into MPR [major pathological response (15)] and Cancer groups based on the proportion of residual tumors. In this classification, pCR was defined as the absence of residual tumor in the primary tumor, MPR as a residual tumor in the primary tumor of 10% or less, and Cancer as a residual tumor in the primary tumor of more than 10%.

### 2.2 Model development

We employed five-fold cross-validation in Cohort 1 and tested only in Cohort 2 using the trained weights from Cohort 1 to evaluate the performance of several mainstream AI models, including CNNs (16–22), Transformers (23–25), and models designed for efficient computation (21, 22, 25). In the five-fold cross-validation, we partitioned the data by the patient rather than by image to ensure that all images from the same patient appeared in only one-fold, thereby preventing potential data leakage. Specifically, for patient-level diagnosis, when the model predicts non-pCR for a particular image, we classify the patient’s overall response as non-pCR. We summarized key design principles to enhance endoscopic AI performance (pretraining, efficient local features, and robust global features) based on the evaluation results. This led to our design of EC-HAENet, a hybrid-architecture ensemble model. EC-HAENet integrates convolutional and attention mechanisms to collectively extract both local and global information from endoscopic images for accurate diagnosis. The overall architecture of EC-HAENet is illustrated in **Figure 1**. Within this architecture, we utilized Dense Convolution Blocks as the local feature extractor and a multi-layer Transformer module with sliding windows as the global feature extractor. Subsequently, the local and global features were concatenated and fed into an ensemble classifier for the final prediction. The ensemble of the local and global features enhanced the ability to identify tumor residue. Two auxiliary classifiers were introduced only during training to facilitate the learning of different branches. Random data augmentation, sharpness-aware minimization (26), and focal loss (27) were used to improve robustness against possible poor image quality and class imbalance. Details of EC-HAENet construction and training hyper-parameters are provided in **Appendix A**.

### 2.3 Statistical analysis

Statistical analysis was conducted with R version 4.41. Differences between groups were examined using the $\chi^{2}$ test. Accuracy, sensitivity, specificity, positive predictive value (PPV), and negative predictive value (NPV) were calculated for Cohort 1 and Cohort 2, and 95% confidence intervals (CI) were calculated using the Clopper-Pearson method. Receiver operating characteristic (ROC) curves were used to calculate the area under the curve (AUC) for Cohort 1 and Cohort 2 to evaluate the diagnostic performance of EC-HAENet. All statistical comparisons were performed using two-tailed tests, and P < 0.05 was considered statistically significant.

## 3 Results

### 3.1 Baseline characteristics

We collected 4,561 images from 209 EC patients as Cohort 1. This cohort included 43 patients (20.6%) with pCR, 45 patients (21.5%) with MPR, and 121 patients (57.9%) with Cancer. Afterward, we formed Cohort 2, which comprised 2,798 images from 91 patients. Within this cohort, 18 patients (19.8%) were pCR, 17 patients (18.7%) were MPR, and 56 patients (61.5%) were Cancer. All patients underwent at least one cycle of TNT, mainly involving chemotherapy and chemoimmunotherapy (n=297, 99.0%), and only 3 patients (1.0%) received preoperative radiotherapy. **Supplementary Table 1** provides more detailed clinical information regarding gender, age, drinking history, tumor site, cT stage, etc.

### 3.2 Diagnostic performance of EC-HAENet

**Figure 2:**
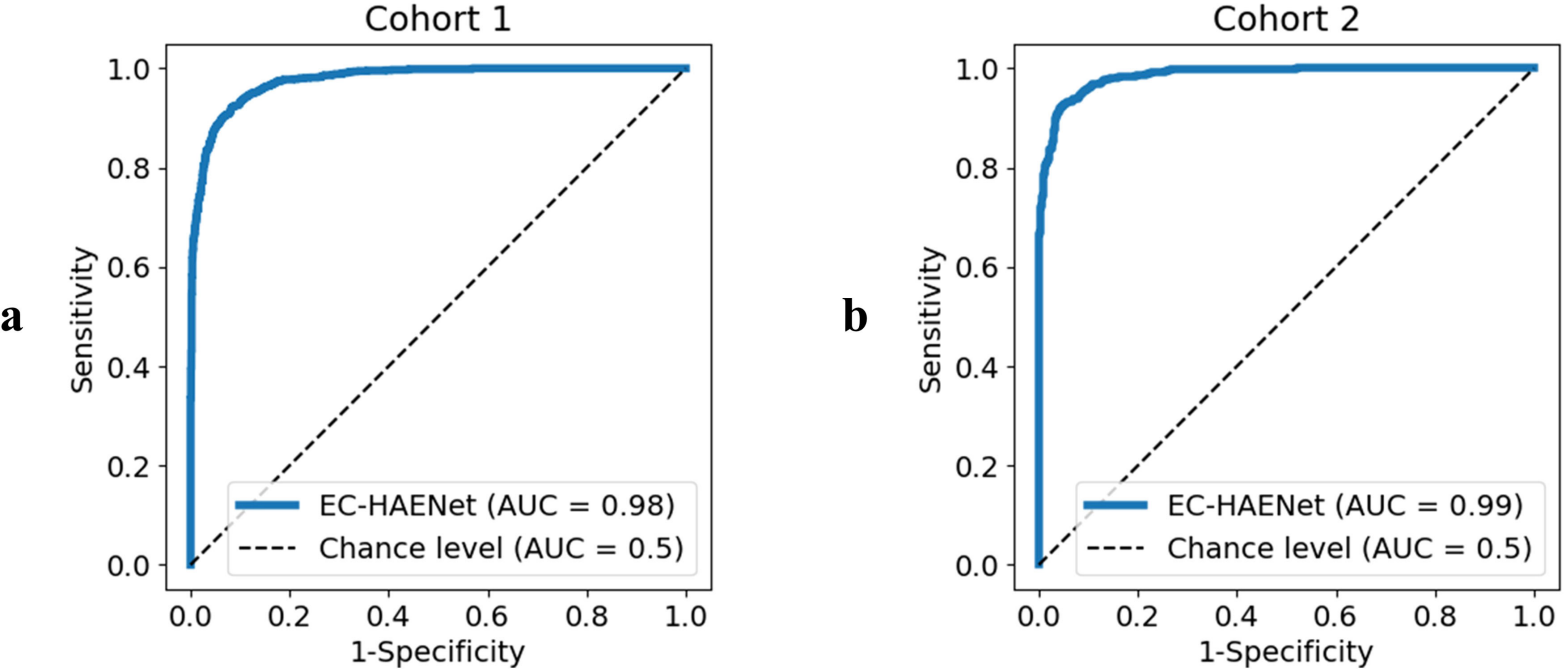
Image-level receiver operating curve of EC-HAENet. **(a)**: Cohort 1, **(b)**: Cohort 2.

As presented in **Table 1** and **Figure 2**, in Cohort 1, our EC-HAENet achieved the highest diagnostic performance in both five-fold cross-validation among all models. The image-level AUC was 0.98 (95%CI: 0.97-0.98), accuracy was 0.92 (0.91-0.93), sensitivity was 0.92 (0.90-0.93), and specificity was 0.92 (0.90-0.93). We externally evaluated its performance in Cohort 2 and found that AUC was 0.99 (0.98-0.99), accuracy was 0.93 (0.92-0.95), sensitivity was 0.95 (0.93-0.96), and specificity was 0.92 (0.90-0.94). For patient-level performance, where we classify the patient’s response as non-pCR when the model predicts non-pCR for a particular image, EC-HAENet achieved an accuracy of 0.93 (0.89-0.96) in Cohort 1 and 0.93 (0.86-0.98) in Cohort 2, the other metrics are also provided in **Table 1** for reference.

**Table 1:** Image-level and patient-level classification metrics of EC-HAENet with 95% confidence interval.

Metrics	Cohort 1		Cohort 2	
	Image-level	Patient-level	Image-level	Patient-level
Accuracy	0.92 (0.91-0.93)	0.93 (0.89-0.96)	0.93 (0.92-0.95)	0.93 (0.86-0.98)
Sensitivity	0.92 (0.90-0.93)	0.98 (0.94-0.99)	0.95 (0.93-0.96)	0.97 (0.90-1.00)
Specificity	0.92 (0.90-0.93)	0.80 (0.66-0.90)	0.92 (0.90-0.94)	0.80 (0.56-0.94)
PPV	0.91 (0.89-0.92)	0.91 (0.78-0.97)	0.94 (0.93-0.96)	0.89 (0.65-0.99)
NPV	0.93 (0.91-0.94)	0.94 (0.89-0.97)	0.93 (0.90-0.94)	0.95 (0.87-0.98)

PPV, positive predictive value; NPV, negative predictive value.

**Figure 3:**
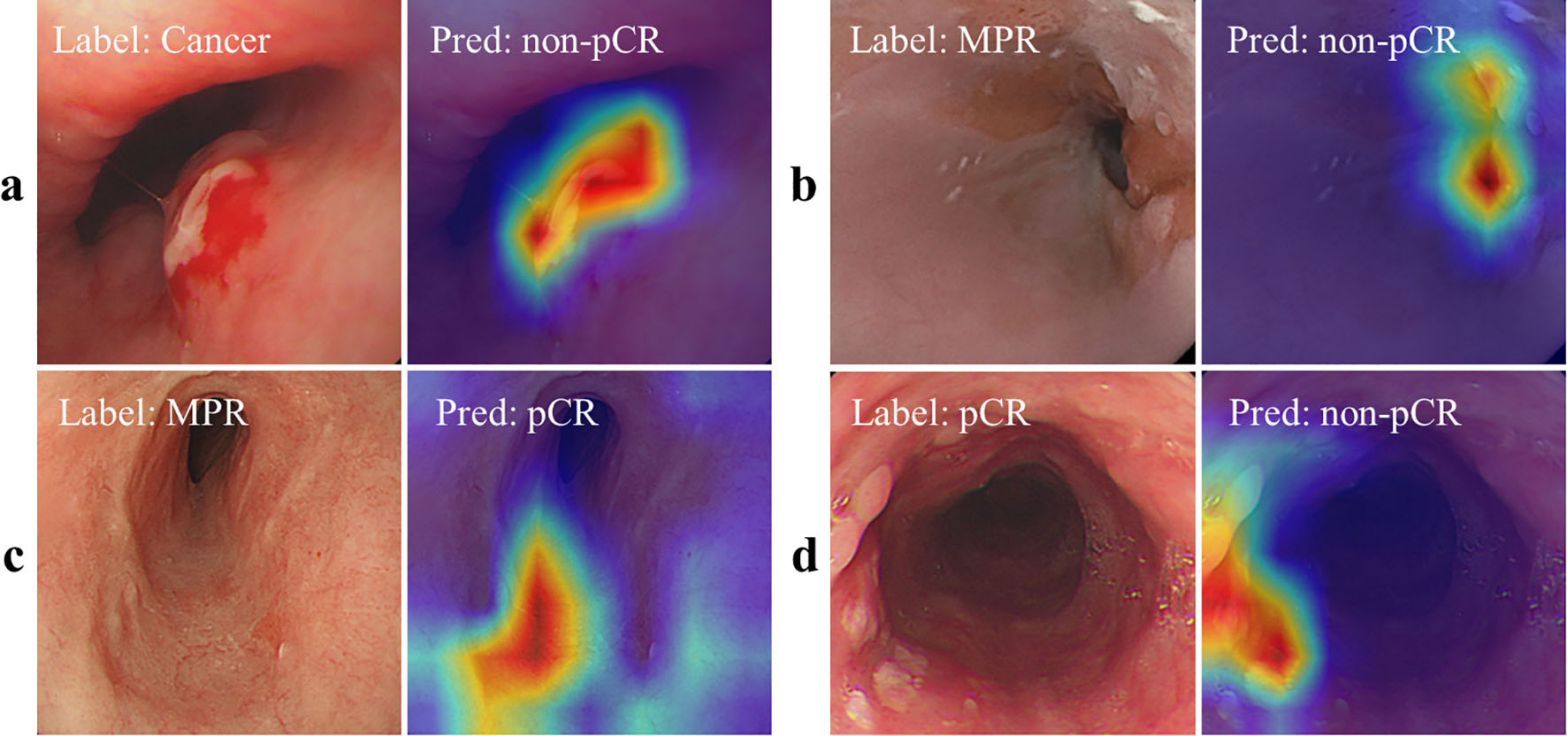
Feature attribution visualization for decision analysis. **(a, b)** are correctly classified images, and the heatmap shows that the model can correctly focus on the lesion areas. **(c)** MPR is incorrectly predicted as pCR. There are no obvious lesion features in the image. **(d)** pCR is incorrectly predicted as cancer. The heatmap shows that the model focuses on the scar areas. pCR, pathological complete response; MPR, major pathological response.

As shown in **Figures 3a, b**, we also used Axiom-based Grad-CAM [XGrad-CAM (28)] to interpret the decisions made by EC-HAENet. The class activation maps (CAMs) visualization results indicated that EC-HAENet could accurately locate the regions containing tumors, achieving reliable image classification. More details for generating the heatmap are provided in **Appendix B**.

### 3.3 Evaluation of different deep learning models

**Figure 4:**
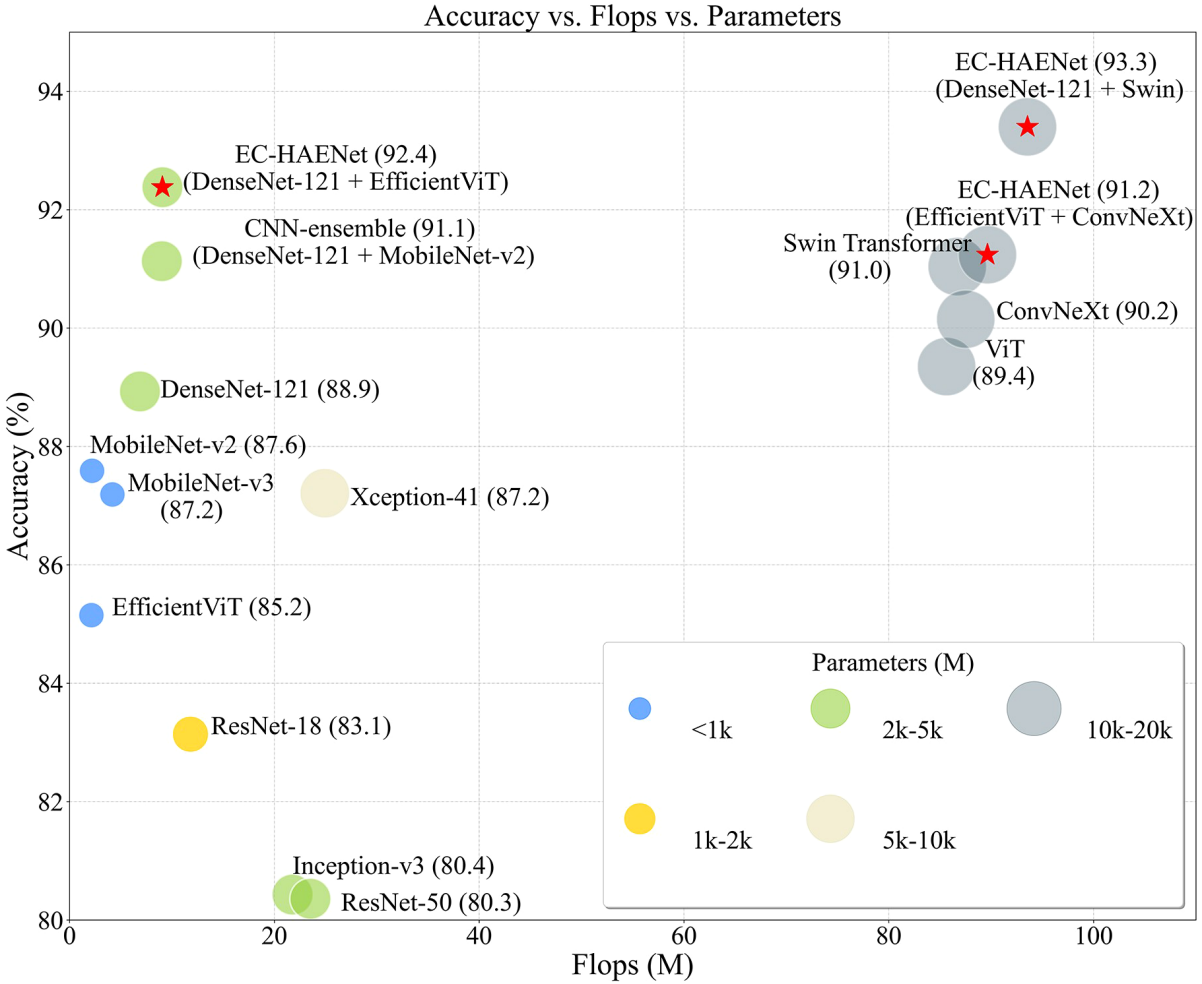
The classification accuracy (in Cohort 2), flops, and parameters of different deep learning models. The numbers in parentheses indicate accuracy. The red stars indicate different EC-HAENet designs with varying parameters and flops. flops, floating-point operations per second.

**Figure 4** illustrates the classification performance of different deep learning models in Cohort 2, with their Flops and model parameters. Flops, or Floating-Point Operations Per Second, is a metric that quantifies a computing system’s performance in executing mathematical calculations and lower Flops mean better computational efficiency. We found that models like Swin Transformer and ConvNeXt, which leverage sliding windows to introduce local bias or expand the receptive field for better long-range dependency, achieved the highest accuracy among existing methods.

EC-HAENet, designed to enhance both local and global feature extraction (using DenseNet-121 as the local CNN block and Swin Transformer as the global attention block), achieved the best classification accuracy (93.3%) across all models without significantly increasing computational load. Alternatively, EC-HAENet can be built with lighter-weight transformer models (such as Efficient ViT) to reduce computational complexity while maintaining superior accuracy compared to other models. For example, ConvNeXt paired with EfficientViT achieved 0.91 accuracy, and DenseNet-121 paired with Efficient ViT achieved 0.92 accuracy.

We further validated the generalization ability of our design principles on other endoscopic image classification tasks on other endoscopic image classification tasks [Kvasir-v2 dataset (29)]. EC-HAENet consistently outperformed mainstream models, with detailed results shown in **Supplementary Figure 1** and **Appendix C**.

### 3.4 EC-HAENet accuracy *vs*. biopsy accuracy

As presented in **Table 2**, in Cohort 1, biopsies were performed on 83 patients during endoscopy before surgery. This part included 50 cases of Cancer, 19 cases of MPR, and 14 cases of pCR. Compared with postoperative pathology, the biopsy has an accuracy of 0.78 (95%CI: 0.68-0.86), a sensitivity of 0.75 (0.64-0.84), and a specificity of 0.93 (0.69-0.99) (**Table 2**). In Cohort 2, 56 patients underwent biopsy, including 33 cases of Cancer, 12 cases of MPR, and 11 cases of pCR. The accuracy of the biopsy was 0.71 (0.59-0.82), sensitivity was 0.64 (0.50-0.77), and specificity was 1.00 (0.74-1.00).

**Table 2:** Patient-level classification metrics of biopsy versus EC-HAENet with 95% confidence interval.

Metrics	Cohort 1		Cohort 2	
	Biopsy	EC-HAENet^1^	Biopsy	EC-HAENet^2^
Accuracy	0.78 (0.68-0.86)	0.93 (0.89-0.96)	0.71 (0.59-0.82)	0.93 (0.83-0.98)
Sensitivity	0.75 (0.64-0.84)	0.98 (0.94-0.99)	0.64 (0.50-0.77)	0.98 (0.88-1.00)
Specificity	0.93 (0.69-0.99)	0.80 (0.66-0.90)	1.00 (0.88-1.00)	0.77 (0.46-0.95)
PPV	0.98 (0.90-1.00)	0.91 (0.78-0.97)	1.00 (0.88-1.00)	0.91 (0.59-1.00)
NPV	0.43 (0.27-0.61)	0.94 (0.89-0.97)	0.41 (0.25-0.59)	0.93 (0.82-0.99)

PPV, positive predictive value; NPV, negative predictive value.

^1^Evaluated by five-fold cross-validation using all Cohort 1 data.

^2^Evaluated using the same data as biopsy in Cohort 2.

Given that the data in Cohort 1 were randomly partitioned into five folds at the patient level for model development and cross-validation, and the data in Cohort 2 were solely utilized for testing, to ensure a fair comparison, we used the average metrics of cross-validation in Cohort 1, and only use the biopsy data in Cohort to compare EC-HAENet to biopsy, the patient-level results are shown in **Table 2**. EC-HAENet demonstrated significantly better diagnostic performance in Cohort 1 (0.93 *vs*. 0.78; accuracy, P<0.0001) and Cohort 2 (0.93 *vs*. 0.71; accuracy, P<0.0001). Other indicators are shown in **Table 2**. Further analysis of MPR patients revealed that the accuracy of biopsy in Cohort 1 and Cohort 2 was 0.53 and 0.58, respectively. EC-HAENet was also significantly superior to biopsy, with an accuracy of 0.79 in Cohort 1 and 0.75 in Cohort 2.

### 3.5 Decision analysis with feature attribution methods

We used the XGrad-CAM method to analyze images misclassified by the EC-HAENet model. The model failed to identify 95 images with residual tumors. With XGrad-CAM analyzing these false negatives, we found that EC-HAENet most misclassified images with poor image quality (n=35) and MPR pathology (n=32) (**Figure 3c**). Additionally, the model incorrectly labeled 38 images without residual tumors. XGrad-CAM analysis of these false positives revealed that EC-HAENet most frequently misclassified images with poor image quality (n=12) and images with scars (n=24). As illustrated in **Figure 3d**, the model erroneously focused on scar areas, resulting in the incorrect classification of pCR as non-pCR.

## 4 Discussion

Our research shows that EC-HAENet can accurately identify responses in patients with EC after TNT. EC-HAENet consistently and reliably performed well in two cohorts. Compared to endoscopic biopsy, EC-HAENet shows higher accuracy in distinguishing between the presence of tumor residue and minimal tumor residue.

Patients who follow the Watch and Wait strategy mainly rely on endoscopy to assess the response of the primary tumor to treatment. However, the accuracy of endoscopy varies depending on the technique and experience of the endoscopist and is highly subjective. The preSANO study showed that the false-negative rate of endoscopic bite-on-bite biopsy with fine-needle aspiration for the primary tumor remained high at 10%-23% (7, 8). Our results resemble those of preSANO. From 2018 to 2024, the biopsy accuracy consistently exceeded 0.7, with sensitivity ranging from 0.64-0.75 and specificity from 0.93-1.00. Nevertheless, the accuracy of MPR remained relatively low, ranging from 0.53-0.58.

AI has demonstrated significant potential in diagnosing EC. In comparison to endoscopists, AI can examine every detail in all images without being affected by distractions or fatigue during endoscopic examinations. A study from China has indicated that AI models can accurately detect early esophageal squamous cell carcinoma with an AUC of 0.95 (95%CI, 0.93-0.97), and endoscopists’ diagnostic accuracy significantly improves after referring to the AI model’s prediction results (30). However, there is limited research on constructing AI models for EC patients after TNT. DAISUKE et al. evaluated four different algorithms using endoscopic images of 98 EC patients from 2004 to 2016, the accuracy, sensitivity, and specificity of four different algorithm models ranged from 0.64-0.81, 0.68-0.81, and 0.37-0.81%, and the best-performing model achieved an AUC of 0.83, falling short of the ideal prediction effect. By contrast, our study demonstrates that the EC-HAENet performed exceptionally well, with an AUC of over 0.98 in both Cohort 1 and Cohort 2. Furthermore, whether judging all images or MPR images, the EC-HAENet’s accuracy surpasses that of biopsy conducted by experienced endoscopists, highlighting the stability and reliability of the EC-HAENet.

We also summarized several design principles for developing better endoscopic AI models from extensive experiments. **Figure 4** illustrates the model accuracy in Cohort 2, parameters, and flops, and we have drawn the following conclusions that may help further refine the model design for accessing pCR after TNT:

1. **Pretraining** is necessary for improving model performance: initializing the model with pre-trained weights from ImageNet-1K (31) can prevent severe overfitting during the training process.
2. **Local features** efficiently access tumor residue: CNNs are more adept at extracting local features and possess stronger inductive bias (32). As shown in **Figure 4**, CNNs achieved competitive model performance with significantly fewer parameters. At the same time, when the number of parameters is comparable (less than 10k), DenseNet-121 demonstrated better classification performance. This may be related to DenseNet-121’s transition layers, which facilitate the propagation of local and low-level features from the shallow layers to the later layers.
3. **Global features** are beneficial for better generalization ability: transformer networks like ViT and Swin Transformer efficiently capture global features. ConvNeXt is also designed to have a large receptive field, leading to better long-range dependency ability. As shown in **Figure 4**, these models perform better than traditional CNNs on the external validation dataset.

There are some limitations in this study. Firstly, the EC-HAENet model was validated using images from a single center, which may not fully capture the broader variability in image quality and characteristics across different devices and endoscopists. Secondly, the model currently lacks the capability to provide real-time diagnostic feedback, which could increase the workload of endoscopists and potentially reduce diagnostic efficiency, particularly for less experienced practitioners. In future work, we aim to integrate the model into examination devices by connecting an external workstation to process the signals before feeding them back to the display screen, enabling real-time assistance to endoscopists and enhancing their ability to identify complex and challenging lesions more effectively. And then perform multicenter external validation to confirm the model’s generalizability and track long-term patient prognosis.

## 5 Conclusion

In summary, we developed the EC-HAENet model and demonstrated superior accuracy and sensitivity in evaluating responses after TNT, outperforming previous AI results and endoscopic biopsy. EC-HAENet’s high efficiency supports the clinical choice of the Watch and Wait strategy in patients post-TNT and the follow-up development potential of the model. Additionally, we identified three key principles for improving endoscopic AI performance in this process: pretraining, efficient local feature extraction, and robust global feature representation. Based on our findings, we hope to ultimately enhance the accuracy of endoscopic biopsy with the assistance of a deep learning model to provide an ethical and technical basis for making non-surgical decisions.

## Data Availability

The original contributions presented in the study are included in the article/**Supplementary Material**. Further inquiries can be directed to the corresponding authors.

## Appendices

A. Details of Model Development.

For the specific details of the model architecture and training, we employed the encoder of DenseNet-121 as the local dense CNN blocks and the encoder of the Swin Transformer (base) as our global attention blocks. The encoders were initialized with weights pre-trained on ImageNet-1k.

During the training phase, to enhance the extraction of both local and global features, we initially introduced two linear auxiliary classifiers consisting of a single fully connected layer. The auxiliary classifiers were used to train the convolutional and attention branches independently. Once both branches had converged, we froze the parameters of the feature extractors in these two branches and proceeded to train only the ensemble classifier.

Random data augmentation including random rotation, random clip-resize random vertical and horizontal flips, and brightness-contrast adjustment was conducted with a probability of 0.3. Focal loss and sharpness-aware minimization were used for model optimization to improve model generalization ability. Different from standard stochastic gradient descent (SGD), which updates the parameters directly along the direction of gradient descent, SAM adds perturbations to the model parameters during training to achieve a flatter loss landscape, leading to better generalization ability as shown in Equation A.1:

(A.1)
$$\begin{array}{c} \underset{w}{\min}E_{\left(x,y\right)\sim D}\underset{\left|\left|\epsilon \right|\right|\leq \rho ;x,y}{\max}L\left(w+\epsilon \right)+\lambda {\left|\left|w\right|\right|}_{2}^{2} \end{array}$$

where D is the data distribution, L is the loss function, w is the parameters of the model, ${\left|\left|w\right|\right|}_{2}^{2}$ is the regularization term and ρ controls the magnitude of weight perturbation.

The learning rate for the pre-trained weights was set to $10^{-4}$ , and $10^{-3}$ for the randomly initialized auxiliary classifier, and the learning rate for the ensemble classifier was set to $10^{-4}$ . The base optimizer used in SAM was SGD, the ϵ was set to 0.1, and the weight decay of SGD optimizer was set to $5e^{-4}$ . During five-fold cross-validation, we random sample fifteen percent of the training data as the dev set to select the best hyper-parameters. The batch size during training was set to 32. The max training epoch for the training encoder was set to 100, and 50 for the ensemble classifier; if the classification performance on the dev set did not improve for 10 epochs, the training process would be stopped early, and the checkpoint with the best classification accuracy and the lowest loss on the dev set would be saved for evaluation.

B. Attribution Methods.

We utilize Axiom-based Grad-CAM (XGrad-CAM) to generate visual explanations for model decisions. Specifically, we average the heatmap generated from the Local Dense CNN Blocks and the Global Attention Blocks as the final feature attribution results. The feature maps from the final convolution layer of the DenseNet-121 and the attention map from the last stage of the Swin Transformer. This integrated approach allows for a more accurate and interpretable understanding of how the model arrives at its decisions, by combining the local and global features represented by the respective blocks and their corresponding maps.

C. Additional Experiments on the Kvasir Dataset.

We also tested different AI models and our EC-HAENet on the Kvasir-v2 dataset to evaluate our summarized principles for AI design for endoscopic image applications. The Kvasir-v2 dataset provides pathological findings labels, including esophagitis, polyps, and ulcerative colitis. It contains 3000 images for pathological findings, and the number of each class is balanced. We random sample 64% data for training, 16% data for validation, and 20% data for testing. The data splits were fixed to compare different models fairly.

We used the same model architecture and training pipeline for developing models on the Kvasir dataset, except for reducing the maximum number of training epochs to 50. The experimental results for Kvasir-v2 are illustrated in **Supplementary Figure 1**. It is worth mentioning that the classification of esophagitis, polyps, and ulcerative colitis is relatively simple, so most existing models can achieve high accuracy. However, EC-HAENet still achieved the highest accuracy of 98.5%, which demonstrated the effectiveness of our proposed design principles.
