# Supplementary figures and images for: Assessing response in endoscopy images of esophageal cancer treated with total neoadjuvant therapy via hybrid-architecture ensemble deep learning
Source: Front Oncol. 2025 May 6;15:1590448. doi: 10.3389/fonc.2025.1590448 (PMC12089136; doi:10.3389/fonc.2025.1590448)

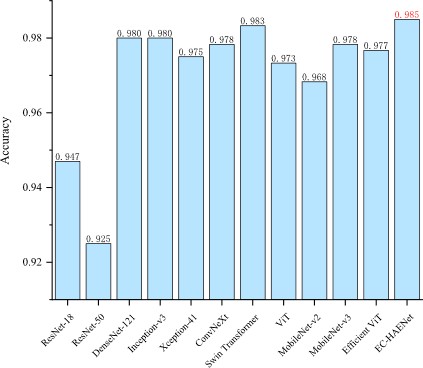

Supplement: Supplementary file 1 [file Image1.jpeg]
