# Supplementary material for: Assessing response in endoscopy images of esophageal cancer treated with total neoadjuvant therapy via hybrid-architecture ensemble deep learning
Source: Front Oncol. 2025 May 6;15:1590448. doi: 10.3389/fonc.2025.1590448 (PMC12089136; doi:10.3389/fonc.2025.1590448)
Supplement: Supplementary file 2 [file Table1.docx]

Supplementary Table 1 Patients baseline and treatment characteristics

| Variables | Total | Cohort 1 | Cohort 2 |
| --- | --- | --- | --- |
|  | n=300(%) | n=209(%) | n=91(%) |
| **Age, years** |  |  |  |
| <60 | 89(29.7) | 63(30.1) | 26(28.6) |
| ≥60 | 211(70.3) | 146(69.9) | 65(71.4) |
| **Gender** |  |  |  |
| Male | 258(86.0) | 177(84.7) | 81(89.0) |
| Female | 42(14.0) | 32(15.3) | 10(11.0) |
| **Drinking** |  |  |  |
| No | 160(53.3) | 134(64.1) | 26(28.6) |
| Yes | 140(46.7) | 75(35.9) | 65(71.4) |
| **Tumor site** |  |  |  |
| Cervical | 7(2.3) | 5(2.4) | 2(2.2) |
| Thoracic | 283(94.4) | 197(94.2) | 86(94.5) |
| EGJ | 10(3.3) | 7(3.4) | 3(3.3) |
| **Histological type** |  |  |  |
| Squamous cell carcinoma | 292(97.3) | 204(97.6) | 88(96.7) |
| Adenocarcinoma | 8(2.7) | 5(2.4) | 3(3.3) |
| **cT** |  |  |  |
| 1 or 2 | 75(25.0) | 57(27.3) | 18(19.8) |
| 3 | 206(68.7) | 138(66.0) | 68(74.7) |
| 4 | 19(6.3) | 14(6.7) | 5(5.5) |
| **cN** |  |  |  |
| 0 | 27(9.0%) | 22(10.5%) | 5(5.5.%) |
| 1~3 | 273(91.0%) | 187(89.5%) | 86(94.5%) |
| **ypT** |  |  |  |
| 0 | 61(20.3) | 43(20.6) | 18(19.8) |
| 1 | 72(24.0) | 57(27.3) | 15(16.5) |
| 2~4 | 167(55.7) | 109(52.1) | 58(63.7) |
| **ypN** |  |  |  |
| 0 | 176(58.7%) | 124(59.3%) | 52(57.1%) |
| 1~3 | 124(41.3%) | 85(40.7%) | 39(42.9%) |
| **Curative effect** |  |  |  |
| pCR | 61(20.3) | 43(20.6) | 18(19.8) |
| MPR | 62(20.7) | 45(21.5) | 17(18.7) |
| Cancer | 177(59.0) | 121(57.9) | 56(61.5) |
| **Type of TNT** |  |  |  |
| Chemotherapy | 91(30.3) | 80(38.3) | 11(12.1) |
| Chemoimmunotherapy | 206(68.7) | 127(60.8) | 79(86.8) |
| Combine RT | 3(1.0) | 2(0.9) | 1(1.1) |
| **Cycle of TNT** |  |  |  |
| 1 or 2 | 227(75.7) | 163(78.0) | 64(70.3) |
| >2 | 73(24.3) | 46(22.0) | 27(29.7) |

*EGJ* esophagogastric junction; *cT* clinical T stage; *cN* clinical N stage; *ypT* pathological T stage after neoadjuvant therapy; *ypN* pathological N stage after neoadjuvant therapy; *pCR* pathological complete response; *MPR* major pathological response; *TNT* total neoadjuvant therapy; *RT* radiotherapy
